# Strange Suggestion

**Published:** 1873-12

**Authors:** 


A Strange Suggestion.—The St. Louis *New Era* makes the following strange suggestion. We hardly think it will be carried into effect. It would be a fatal advertisement for some M.D.'s : "In marriage notices it is usual to give the name of the clergyman who performed the ceremony ; and with equal propriety, in obituary notices, the name of the attending physician should be given."—*The Doctor, Nov.* 1, 1873.

